# Inactivation of SARS-CoV-2 Laboratory Specimens

**DOI:** 10.4269/ajtmh.21-0229

**Published:** 2021-04-20

**Authors:** Elaine Haddock, Friederike Feldmann, W. Lesley Shupert, Heinz Feldmann

**Affiliations:** 1 Laboratory of Virology, Division of Intramural Research, National Institute of Allergy and Infectious Diseases, National Institutes of Health, Hamilton, Montana;; 2 Rocky Mountain Veterinary Branch, Division of Intramural Research, National Institute of Allergy and Infectious Diseases, National Institutes of Health, Hamilton, Montana

## Abstract

The burden on diagnostic and research laboratories to provide reliable inactivation for biological specimens to allow for safe downstream processing is high during the coronavirus disease 2019 (COVID-19) pandemic. We provide safety data regarding commonly used chemical and physical inactivation procedures that verify their effectiveness against severe acute respiratory syndrome coronavirus 2 (SARS-CoV-2).

Severe acute respiratory syndrome coronavirus 2 (SARS-CoV-2) is the cause of the current coronavirus disease 2019 (COVID-19) pandemic.^1–3^ There is a high demand for safe sample handling to perform tests on in vitro and in vivo material in research and diagnostic laboratories worldwide. SARS-CoV-2 is considered a risk group 3 pathogen, and safety at workplaces is of the highest priority.^4^ SARS-CoV-2 is an enveloped, single-strand, positive-sense RNA virus with a genome size of approximately 30 kb.^2,3,5^ Its biological, biochemical, and physical features make the virus sensitive to chemical and physical inactivation procedures. We evaluated commonly used inactivation procedures to generate safe material for downstream genome, protein, immune response, and histopathology analyses.

All infectious work was performed under high biocontainment conditions at the Rocky Mountain Laboratories of the National Institute of Allergy and Infectious Diseases (NIAID) according to standard operating protocols (SOPs) approved by the Institutional Biosafety Committee (IBC). For our studies, we used the SARS-CoV-2 isolate nCoV-WA1-2020 (MN985325.1; kindly provided by the Centers for Disease Control and Prevention).^6^ SARS-CoV-2 replication in cell culture causes a cytopathic effect (CPE), thus allowing for a simple readout parameter. SARS-CoV-2 stocks were grown in VeroE6 cells and titrated using a tissue culture infectious dose 50% (TCID_50_) assay.^7^ The TCID_50_ was calculated via the Reed-Muench formula to a concentration of 4 × 10^6^ TCID_50_/mL.^8^ Cells were produced by infecting VeroE6 cells at a multiplicity of infection of 0.01 SARS-CoV-2. Cells were harvested in Dulbecco's phosphate-buffered saline (DPBS) at CPE of approximately 75%, counted, and frozen (−80°C) in aliquots of 2 × 10^6^, 5 × 10^6^, and 2 × 10^7^ cells/mL. SARS-CoV-2-infected lung tissue (≈1 × 10^10^ TCID_50_/g) was obtained from a previous Syrian hamster study approved by the Institutional Animal Care and Use Committee.^9^

We tested the physical and chemical inactivation of virus stocks as well as chemical inactivation of infected cells and tissue. Triplicate samples were dialyzed with an 8- to 10-kDa molecular weight cutoff (Repligen Corporation, Waltham, MA) using DPBS over a stir plate at 4°C (> 500-fold exchange volumes, five changes during 32–48 hours) or run over a detergent removal column (DetergentOUT GBS10-5000 columns; G-Biosciences, St. Louis, MO). DPBS and noninfected VeroE6 cells and hamster lung tissues served as negative controls. Untreated virus stocks and SARS-CoV-2-infected VeroE6 cells and hamster lung tissue were used as positive controls. All samples were brought to a final volume of 3 ml and equally divided to infect VeroE6 cells (80% confluency) in triplicate for a total of 9 flasks per sample type. Cells were incubated at 37°C for 7 days and monitored regularly for CPE. Three days after initial infection, samples were passaged by transferring 1 mL supernatant each to a new flask of VeroE6 cells (80% confluency). Additionally, we tested extracted RNA with or without transfection reagents to assess RNA infectivity. The results are summarized in Table 1, and detailed protocols are provided as Supplementary Material.

**Table 1:** Summary of inactivation methods and results

Inactivation method	Reagent volume	Sample type*	Inactivated sample (final viral load)	Contact time	Temp.	Reagent removal process	Result (initial infection)	Result (passage)
Irradiation	1 mL	Liquid virus stock	1 × 10^6^ TCID_50_	0, 0.2, 0.4, 0.6, or 0.8 Mrd dose	Dry ice		Positive (9/9)	Positive (9/9)
	1 mL	Liquid virus stock	1 × 10^6^ TCID_50_	1.0 Mrd dose	Dry ice		Negative (0/9)	Negative (0/9)
Buffer AVL + ethanol†	560 μL	Liquid virus stock	140 μL (5.6 × 10^5^ TCID_50_)	10 min + 20 min	20°C	Dialysis	Negative (0/9)	Negative (0/9)
Buffer AVL RNA extract transfection	560 μL	Liquid virus stock RNA extract	30 μL RNA extract (2.8 × 10^5^ TCID_50_ equivalent)	10 min + 20 min	20°C	Extraction	Negative (0/6)	Negative (0/6)
	560 μL	Liquid virus stock RNA extract with TransIT LT1	30 μL RNA extract (2.8 × 10^5^ TCID_50_ equivalent)	10 min + 20 min	20°C	Extraction	Negative (0/6)	Negative (0/6)
	560 μL	Liquid virus stock RNA extract with Lipofectamine LTX	30 μL RNA extract (2.8 × 10^5^ TCID_50_ equivalent)	10 min + 20 min	20°C	Extraction	Negative (0/6)	Negative (0/6)
	560 μL	Liquid virus stock RNA extract with TransIT mRNA	30 μL RNA extract (2.8 × 10^5^ TCID_50_ equivalent)	10 min + 20 min	20°C	Extraction	Positive (6/6)	Positive (6/6)
Buffer RLT + ethanol†	600 μL	Cell pellet	5 × 10^6^ infected cells (∼5 × 10^6^ TCID_50_)	10 min + 20 min	20°C	Dialysis	Negative (0/9)	Negative (0/9)
	600 μL	Tissue	30 mg (∼3 × 10^8^ TCID_50_)	10 min + 20 min	20°C	Dialysis	Negative (0/9)	Negative (0/9)
Trizol	275 μL	Liquid virus stock	125 μL (5 × 10^5^ TCID_50_)	10 min	20°C	Dialysis	Negative (0/9)	Negative (0/9)
	300 μL	Cells in 150 µL	5 × 10^6^ infected cells (∼5 × 10^6^ TCID_50_)	10 min	20°C	Dialysis	Negative (0/9)	Negative (0/9)
	600 μL	Tissue	50 mg (∼5 × 10^8^ TCID_50_)	10 min	20°C	Dialysis	Negative (0/9)	Negative (0/9)
Formalin	1 mL	Cells	2 × 10^6^ infected cells (∼2 × 10^6^ TCID_50_)	Overnight	4°C	Dialysis	Negative (0/9)	Negative (0/9)
	10 mL, 10% final	Tissue	1.4 g (∼1.4×^10^ TCID_50_)	7 days	4°C	Dialysis	Negative (0/9)	Negative (0/9)
Paraformaldehyde	1 mL, 2% final	Cells	2 × 10^7^ infected cells (∼2 × 10^7^ TCID_50_)	Overnight	4°C	Dialysis	Negative (0/9)	Negative (0/9)
	10 mL, 2% final	Tissue	0.7 g (∼7 × 10^9^ TCID_50_)	7 days	4°C	Dialysis	Negative (0/9)	Negative (0/9)
1% SDS^‡^	100 μL, 4×	Liquid virus stock	300 µL (1.2 × 10^6^ TCID_50_)	10 min	100°C^§^	Detergent column	Negative (0/9)	Negative (0/9)
	100 μL, 4×	Cells in 300 μL	5 × 10^6^ infected cells (∼5 × 10^6^ TCID_50_)	10 min	100°C§	Detergent column	Negative (0/9)	Negative (0/9)

Mrd = Megarad.

* Initial sample viral titers were as follows: liquid SARS-CoV-2 virus stock (4 × 10^6^ tissue culture infectious dose 50% [TCID_50_]/mL); infected cells (∼1 × 10^7^ TCID_50_/mL, 1 × 10^7^ cells/mL); and tissue (∼10^10^ TCID_50_/g). Infected cells were pelleted by centrifugation and lysed directly in Buffer RLT or pelleted and resuspended in Dulbecco's phosphate-buffered saline at the concentration and volumes described in the Table before addition of other test reagents). Lung tissue was collected from Syrian hamsters at the height of infection with SARS-CoV-2 or from mock-infected hamsters on a corresponding day.

† Addition of ethanol: after contact time with buffer AVL or buffer RLT, samples were transferred to a clean tube with 560 μL 100% ethanol (for AVL inactivation) or 600 μL 70% ethanol (for RLT inactivation) and allowed an additional 20 minutes of contact time at 20°C.

‡ 4 × SDS loading buffer contains 200 mM Tris (pH 6.8), 4% SDS, 35% glycerol, 0.05% bromophenol blue, and 20% 2-ME (added at the time of use).

§ Samples were boiled in an AccuBlock Digital Dry Bath.

## IRRADIATION

Inactivation by irradiation is the preferred method for specimens used for a variety of serological, immunological, and biochemical assays, for which authentic protein and particle structure are critical. We previously determined that a dose of 1.0 Megarad (10,000 Gray) completely inactivated SARS-CoV-1, strain Tor 2 *(10)*. To determine a breakthrough dose for inactivation, we treated 1 × 10^6^ TCID_50_ of SARS-CoV-2 with 0, 0.2, 0.4 0.6, 0.8, and 1.0 Megarad. We used a JL Shepherd Model 484R irradiator (JL Shepherd and Associates, San Fernando, CA) using a cobalt-60 source as previously described.^10^ Irradiation was performed on dry ice and samples were co-located with lithium fluoride film dosimeters on a rotating platform to assure homogeneous irradiation and confirm the absorbed doses. VeroE6 cells were infected in triplicate with the treated samples and monitored for CPE. As with SARS-CoV-1, only the dose of 1.0 Megarad completely inactivated SARS-CoV-2 (Table 1).

## CHAOTROPIC REAGENTS

For nucleic acid extraction, we used the guanidinium isothiocyanate buffers AVL and RLT (Qiagen, Hilden, Germany) as well as TRIzol (Life Technologies, Carlsbad, CA), as recommended by the manufacturers. AVL was mixed with stock virus at a ratio of 1:5 (sample:total) and incubated for 10 minutes at room temperature, followed by transfer to 560 μL of 100% ethanol for 20 minutes at room temperature (Table 1). Infected cells (5 × 10^6^) were resuspended and incubated in RLT for 10 minutes, followed by the addition of 600 μL of 70% ethanol for an incubation period of 20 minutes. Infected lung tissue was homogenized in RLT with a stainless-steel bead (10 minutes at 30 Hz). A soluble aliquot (equivalent to ≈30 mg) was transferred to a new tube and fresh RLT was added to a volume of 600 µl, followed by the addition of 70% ethanol (600 μL) for 20 minutes. TRIzol was mixed with stock virus at a ratio of 1:3.2 (sample:total) and incubated for 10 minutes at room temperature (Table 1). Infected cells (5 × 10^6^) were resuspended and treated with TRIzol (1:3 sample:total). Infected lung samples were homogenized, and an aliquot of tissue homogenate (≈50 mg) was transferred to a new tube and fresh TRIzol was added. After immediate dialysis, VeroE6 cells were inoculated with the treated material and monitored for CPE. In all cases, no CPE was observed, indicating complete inactivation of SARS-CoV-2 by AVL, RLT, and TRIzol (Table 1).

## RNA INFECTIVITY

The single-strand, positive-sense RNA genome of coronaviruses may start a replication cycle on entry into cells, subsequently generating infectious virus. Therefore, we performed direct infectivity testing of extracted RNA in water on VeroE6 cells. RNA was simply added to the cell monolayer or transfected with the reagents Lipofectamine LTX (Invitrogen, Carlsbad, CA), TransIT LT1 (Mirus Bio, Madison, WI), or TransIT-mRNA (Mirus Bio) according to the manufacturers’ recommendations. Although TransIT LT1 is designed primarily for DNA transfection and unlikely to aid the uptake of the SARS-CoV-2 RNA genome, Lipofectamine LTX supports transfection of both RNA and DNA components, and TransIT-mRNA is specifically designed for RNA transfection. Transfection mixtures containing 30 μL RNA (one-half the elution volume) were tested in duplicate, and each sample was evenly split across three wells of a six-well plate of VeroE6 cells at 80% confluency. No CPE was observed by viral RNA alone or transfection with reagents other than TransIT-mRNA, indicating that SARS-CoV-2 genomic RNA is noninfectious under these conditions (Table 1).

## FIXATIVES

Formalin and paraformaldehyde are commonly used to fix cells or tissues for histologic or microscopic analyses. Infected cells (2 × 10^6^) were diluted in 1 mL 10% neutral-buffered formalin (Leica Biosystems, Wetzlar, Germany) or (2 × 10^7^) 2% paraformaldehyde (Electron Microscopy Services, Hatfield, PA) overnight at 4°C. Samples were pelleted by centrifugation and washed with DPBS in a minimum of three cycles to remove fixative and used to infect VeroE6 cells. Monitoring of cells revealed the absence of CPE, indicating complete inactivation of SARS-CoV-2 (Table 1).

Infected hamster tissue was incubated in 10 mL of 10% neutral-buffered formalin (full lungs) or 2% paraformaldehyde (half lung) for a period of 7 days at 4°C. Subsequently, a small section of tissue (≈150 mg) was dissected and homogenized in DPBS. After a minimum of three cycles of pelleting and washing with DPBS, samples were used to infect VeroE6 cells. Monitoring of cells confirmed the absence of CPE, indicating complete inactivation of SARS-CoV-2 (Table 1).

## DETERGENTS

Detergent treatment under heat is often used for protein analysis. Aliquots of virus stock (300 μL) or infected cells (5 × 10^6^ in 300 μL) were diluted in 100 μL 4 × loading buffer (1% sodium dodecyl sulfate [SDS] final) and boiled at 100°C for 10 minutes. Detergent was immediately removed through DetergentOUT columns (G-Biosciences). Treated samples were tested for inactivation on VeroE6 cells. Although titers were not measured from control samples after this treatment, CPE via the positive samples was not delayed, indicating that a significant reduction in virus because of treatment alone was unlikely. In test samples, no CPE was noticed, indicating complete inactivation of SARS-CoV-2 (Table 1).

## CONCLUSIONS

The study verified the safe use of common chemical and physical treatment procedures for the complete inactivation of SARS-CoV-2 in distinct specimen types (Table 1). Additionally, we have shown that SARS-CoV-2 genomic RNA alone is unlikely to spontaneously generate virus from introduction to cells. The inactivation procedures are likely to apply to all coronaviruses because of the similar biological, biochemical, and biophysical features within the virus family. These data may provide guidance for IBCs to evaluate SOPs for the inactivation of SARS-CoV-2 containing biological specimens. The well-defined results may also assist in the improvement and approval of SOPs for inactivation without the need to verify inactivation for individual samples, which is unfeasible with the current diagnostic and research operations.
